# Metallothioneins 1 and 2, but not 3, are regulated by nutritional status in rat white adipose tissue

**DOI:** 10.1186/s12263-016-0533-3

**Published:** 2016-06-23

**Authors:** Sylwia Szrok, Ewa Stelmanska, Jacek Turyn, Aleksandra Bielicka-Gieldon, Tomasz Sledzinski, Julian Swierczynski

**Affiliations:** 1 Department of Biochemistry, Medical University of Gdansk, Debinki 1, 80-211 Gdansk, Poland; 2 Department of Environmental Technology, University of Gdansk, Wita Stwosza 63, 80-952 Gdansk, Poland; 3 Department of Pharmaceutical Biochemistry, Medical University of Gdansk, Debinki 1, 80-211 Gdansk, Poland

**Keywords:** Metallothioneins, Insulin, cAMP, Fasting, WAT

## Abstract

**Background:**

Cumulating evidence underlines the role of adipose tissue metallothionein (MT) in the development of obesity and type 2 diabetes. Fasting/refeeding was shown to affect MT gene expression in the rodent liver. The influence of nutritional status on MT gene expression in white adipose tissue (WAT) is inconclusive. The aim of this study was to verify if fasting and fasting/refeeding may influence expression of MT genes in WAT of rats.

**Results:**

Fasting resulted in a significant increase in MT1 and MT2 gene expressions in retroperitoneal, epididymal, and inguinal WAT of rats, and this effect was reversed by refeeding. Altered expressions of MT1 and MT2 genes in all main fat depots were reflected by changes in serum MT1 and MT2 levels. MT1 and MT2 messenger RNA (mRNA) levels in WAT correlated inversely with serum insulin concentration. Changes in MT1 and MT2 mRNA levels were apparently not related to total zinc concentrations and MTF1 and Zn transporter mRNA levels in WAT. Fasting or fasting/refeeding exerted no effect on the expression of MT3 gene in WAT. Addition of insulin to isolated adipocytes resulted in a significant decrease in MT1 and MT2 gene expressions. In contrast, forskolin or dibutyryl-cAMP (dB-cAMP) enhanced the expressions of MT1 and MT2 genes in isolated adipocytes. Insulin partially reversed the effect of dB-cAMP on MT1 and MT2 gene expressions.

**Conclusions:**

This study showed that the expressions of MT1 and MT2 genes in WAT are regulated by nutritional status, and the regulation may be independent of total zinc concentration.

**Electronic supplementary material:**

The online version of this article (doi:10.1186/s12263-016-0533-3) contains supplementary material, which is available to authorized users.

## Background

Metallothioneins (MTs) constitute the family of cysteine- and metal-rich, low molecular mass (61 to 68 amino acid) single-chain proteins [41, 47]. Four MT isoforms, MT1, MT2, MT3, and MT4, were found in mammals [47]. MT1 and MT2 are predominant isoforms expressed in many animal tissues. MT1 and MT2 expression is induced by several factors including metals, some drugs, glucocorticosteroids, oxidative stress, and inflammatory mediators [47]. MT3 (also referred to as growth inhibitory factor (GIF)) and MT4 genes are expressed in the brain (predominantly in Zn-containing neurons of the hippocampus, pineal gland, and retina) and stratified squamous epithelial cells [1, 14, 47]. Expression of MT3 was also found in some peripheral organs of rats [20]. Although MT3 and MT4 are often referred to as non-inducible proteins [47], some data imply that also MT3 may be, at least in part, an inducible protein, and its expression is modulated by metal concentrations [14]. While all MT isoforms show similar metal binding capacities, they likely have different biological functions. Available evidence suggests that MT1 and MT2 may be involved in (a) protection against heavy metal- and reactive oxygen species-induced toxicity [36], (b) Zn and Cu homeostasis, as they sequester and/or dispense Zn and Cu [40], and (c) acute phase response [4]. Noticeably, however, mice with disrupted MT1 and MT2 genes were viable and developed normally under standard conditions, despite greater susceptibility to cadmium toxicity [31]. Moreover, it has been suggested that the increased level of MT1 in mice might have an important role in reducing morbidity in old animals [29]. Recent studies imply that both MT1 and MT2 may influence mammal longevity [21]. Moreover, MTs were postulated to play a role in the prevention of cardiomyopathy [5, 6] and intermittent hypoxia-induced renal injury [50]. Relatively little is known about the biological functions of MT3 and MT4. MT3 knockout mice developed normally under standard conditions but were more susceptible to kanic acid-induced seizures and showed a greater degree of resultant hippocampal neuronal injury [15]. The potential role of MT3 in the progression of neurodegenerative diseases still raises some controversies; while according to some authors MT3 acts as a protective factor against neurodegenerative diseases, others showed that it may stimulate progression thereof [1, 14, 19, 22]. The evidence from in vivo studies suggests that ectopic expression of MT3 encoding gene stimulates pancreatic acinar necrosis; however, the molecular basis for this toxic effect is unknown [34]. MT4 is likely involved in the regulation of Zn metabolism during the differentiation of epithelial cells [33].

The list of tissues/organs that express MTs encoding genes includes also brown and white adipose tissue [3, 43, 45]. Some evidence from animal studies implies that MT1 and MT2 may be involved in the regulation of energy balance [2]. Furthermore, these MTs may play a role in the prevention of high-fat diet-induced obesity [37]. Moreover, human studies documented enhanced expression of MT2a encoding gene in adipose tissue from an obese subject [7, 11] and patients with type 2 diabetes [16]. This implies that the upregulation of MT gene expression in human adipose tissue may exert a detrimental effect promoting obesity or be a consequence of obesity. In view of data mentioned above, further studies are needed to establish whether MTs really play a role in the etiopathogenesis of obesity and type 2 diabetes. Both these conditions are strongly influenced by the amount of consumed food [18] and intracellular zinc homeostasis [38, 42, 46, 48]. Among other factors, intracellular zinc homeostasis is regulated by MTs [8, 26, 30]. It has been shown that overexpression of MTs quickly diminishes intracellular zinc concentration in cell culture [28]. Thus, it is likely that overexpression of genes coding MT in vivo could decrease zinc concentration in adipocytes, and subsequently biosynthesis of zinc finger proteins, which are the important transcription factors regulating adipogenesis, obesity, and related diseases [48].

Higashimoto et al. [17] and Kondoh et al. [25] found that the liver content of MTs in fasted mice increases significantly, perhaps due to starvation-stimulated synthesis of MTs. In contrast, fasting and refeeding exert no effect on MT1 gene expression in epididymal white adipose tissue of mice [43, 45]. Surprisingly, a significant increase in MT gene expression was observed in a primary culture of rat adipocytes incubated with cAMP or insulin [44]. The abovementioned discrepancies between the results of in vivo (especially, no effect of fasting and fasting/refeeding on MT gene expression in white adipose tissue (WAT)) and in vitro studies (stimulatory effect of cAMP and insulin on MTs genes in primary adipocyte culture) require further clarification.

The aim of this study was to analyze factors that potentially may regulate expression of genes coding MTs in rat WAT. We concentrated especially on the effects of fasting and fasting/refeeding on MT gene expression in main fat depots of rats, as well as an effect of insulin and cAMP on MT messenger RNA (mRNA) levels in isolated adipocytes.

## Methods

### Animals and treatment, tissue and blood collection

The experiment included 10-week-old male Wistar rats. The animals were housed at 22 °C in individual wire-mesh cages, under a 12:12 h light to dark cycle with lights on at 7:00 a.m. The rats were randomly assigned to seven groups (10 animals each): (a) control (ad libitum-fed animals with free access to food and tap water); (b) fasted for 24, 48, or 72 h, and (c) fasted for 48 h and then refed ad libitum for 12, 24, or 48 h.

The rats were killed by decapitation (between 8:00 and 10:00 a.m.). White adipose tissue: retroperitoneal, epididymal, and inguinal, as well as liver and esophagus, were harvested, weighed, and frozen in liquid nitrogen immediately upon collection and stored at −80 °C until analysis. Blood samples were obtained from the cervical artery. After 1 h, the blood cells were removed by centrifugation at 1500×*g* for 10 min. Obtained serum samples were stored at −80 °C until analysis.

All institutional and national guidelines for the care and use of laboratory animals were followed.

The study was approved by the Local Ethics Committee for Experimental Animals in Gdansk, Poland (14/2012).

### Isolation and primary culture of rat adipocytes

Adipocytes from epididymal WAT of 10-week-old male rats were isolated by collagenase digestion as described by [35]. The tissue was placed in polypropylene tubes with Krebs-Ringer buffer (37 °C) containing 1 % bovine serum albumin suitable for cell culture (Sigma-Aldrich, USA), 5.5-mM glucose, 20-mM HEPES (pH 7.6), and 1-mg/ml collagenase (type II; Sigma-Aldrich, USA). The tissue was finely cut with scissors and incubated at 37 °C for 1 h with continuous shaking. After the incubation, the tissue was filtered using 180-μm nylon filters. Adipocytes were washed three times with Krebs-Ringer buffer. The adipocytes isolated from each rat were divided into equal portions and transferred onto incubation plate (Corning, USA) with 2-ml Dulbecco’s modified Eagle’s medium (DMEM) (Sigma-Aldrich, USA) with 5.5-mM glucose supplemented with 1 % bovine serum albumin. The adipocytes were incubated at 37 °C (under 5 % CO_2_ plus 95 % O_2_) for 24 h: (a) without addition (control) or (b) in the presence of insulin (10 μg/ml), (c) forskolin (12 μM) (Sigma-Aldrich, USA), (d) 0.2-mM dibutyryl-cAMP (dB-cAMP) (Sigma-Aldrich, USA), and (e) insulin (10 μg/ml) plus dB-cAMP (0.2 mM).

### RNA isolation

Total RNA (from rat adipose tissue and liver, as well as from isolated adipocytes) was extracted using the Purezol reagent (Bio-Rad, USA) according to the manufacturer’s protocol. Concentration of the RNA was determined on the basis of absorbance at 260 nm. All samples had 260/280 nm absorbance ratio of approximately 2.0.

### Reverse transcription

First-strand complementary DNA (cDNA) was synthesized from 4 μg of total RNA (RevertAidTM First Strand cDNA Synthesis Kit; Thermo Scientific, USA). Prior to amplification of cDNA, each RNA sample was treated with RNase-free DNase I (Thermo Scientific, USA) at 37 °C for 30 min.

### Real-time PCR

Real-time PCR amplification was performed in a 20-μl volume using iQ SYBR Green Supermix (Bio-Rad, USA). Each reaction mix contained cDNA and 0.3 μM of each primer. Primers were designed using Primer-BLAST software (NCBI, USA) and synthesized at Genomed (Poland). Forward and reverse primer sequences are presented in supplementary material (Additional file 1: Table S1). The samples were incubated at 95 °C for 5 min to obtain an initial denaturation and polymerase activation, followed by 35 PCR cycles of amplification (92 °C for 20 s, 57 °C for 20 s, and 72 °C for 40 s). Control reactions, with omission of the RT step or with no template cDNA added, were performed for each assay. All the samples were run in triplicate. To adjust for variations in the amount of added RNA and efficiency of the reverse transcription, β-actin, cyclophilin, and TBP mRNAs were quantified in corresponding samples of WAT and liver, and the results were normalized to these values. Since the results regarding MT mRNA levels in WAT and liver obtained with β-actin, cyclophilin, and TBP were essentially similar, we presented the relative gene expression only as precisely corresponding mRNA/β-actin mRNA in the “Results” section. Relative quantities of the transcripts were calculated using the 2^−ΔΔCT^ formula [27]. The results are expressed in arbitrary units, with one unit corresponding to mean mRNA level for the control group. Amplification of specific transcripts was further confirmed by obtaining melting curve profiles and subjecting the amplification products to 1 % agarose gel electrophoresis.

### Western blots

Western blots were performed as recently described [39]. Briefly, frozen liver or adipose tissue were homogenized in 20-mM Tris-HCl buffer (pH 7.8) containing 0.2 % Triton X-100 and protease inhibitor cocktail (Sigma-Aldrich, USA) and centrifuged (30 000×*g* for 20 min). Aliquots of the supernatants containing 20-μg protein (estimated by Lowry’s method) and the molecular mass protein markers were separated by 10 % SDS-PAGE and electroblotted to Immun-Blot PVDF Membrane (Bio-RAD, USA). The membranes were blocked with 5 % albumin in phosphate buffered saline (PBS) with 0.05 % Tween 20 (Sigma-Aldrich, USA). Subsequently, the membranes were incubated with antibodies diluted in blocking buffer. Monoclonal mouse antibodies against both MT1 and MT2 (MT1/2) (UC1MT) were purchased from Abcam (GB). Anti-actin (A5060), as well as HRP-conjugated secondary anti-rabbit (A0545) and anti-mouse antibodies (A9044), was obtained from Sigma-Aldrich (USA). The bands were visualized using ChemiDoc XRS (Bio-Rad, USA) and compared with respective molecular mass protein markers (SM26634) obtained from Thermo Scientific (USA), visualized on the membrane after electroblotting.

Serum samples were diluted with 20-mM Tris-HCl buffer (pH 7.8), and aliquots of the diluted solution containing 20-μg serum protein (protein estimated by Lowry’s method) were separated by 10 % SDS-PAGE and electroblotted to Immun-Blot PVDF Membrane (Bio-RAD, USA). Further steps of western blots were performed as described above for liver or adipose tissue samples.

### Serum insulin concentration assay

Serum insulin concentrations were determined by enzyme-linked immunosorbent assay (ELISA) (RAI008R; Biovendor, Czech Republic).

### Determination of total zinc levels in adipose tissues

The total amount of Zn in adipose tissues was determined by flame atomic absorption spectrometry (spectrometer AAnalyst 400 Perkin Elmer) after dry ashing at 450 °C. Approximately 0.3 g of epididymal adipose tissue was combusted with gradual increase of temperature (50 °C/h). The obtained ash was treated with 5 ml of hydrochloric acid (6 M), and then, the acid solution was evaporated to dryness. The obtained residue was dissolved in 5 ml of nitric acid (0.1 M) and used for zinc determination at 213.9 nm. All analyses were done in triplicate. The accuracy and precision of the used method was assured by simultaneous analysis of certified reference material M-3 HerTis (Herring Tissue).

### Statistics

Statistical significance of intergroup differences was verified on the basis of one-way analysis of variance (ANOVA) with Tukey’s post hoc test. If data were not normally distributed, they were verified by non-parametric Kruskal–Wallis test. Pearson’s correlation coefficient was calculated to assess the correlation between body weight or adipose tissue mass and relative MT mRNA level. All results are presented as means ± standard deviations (SD). The differences were considered significant at *p* < 0.05. Sigma Stat software was used for all statistical analyses.

## Results

**Fig. 1:**
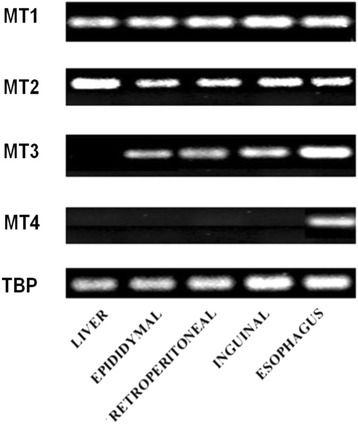
Detection of MT1, MT2, MT3, and MT4 mRNA in the liver, main pads of WAT (epididymal, retroperitoneal, inguinal), and the esophagus of rats by real-time PCR analysis. TBP-TATA-binding protein

**Fig. 2:**
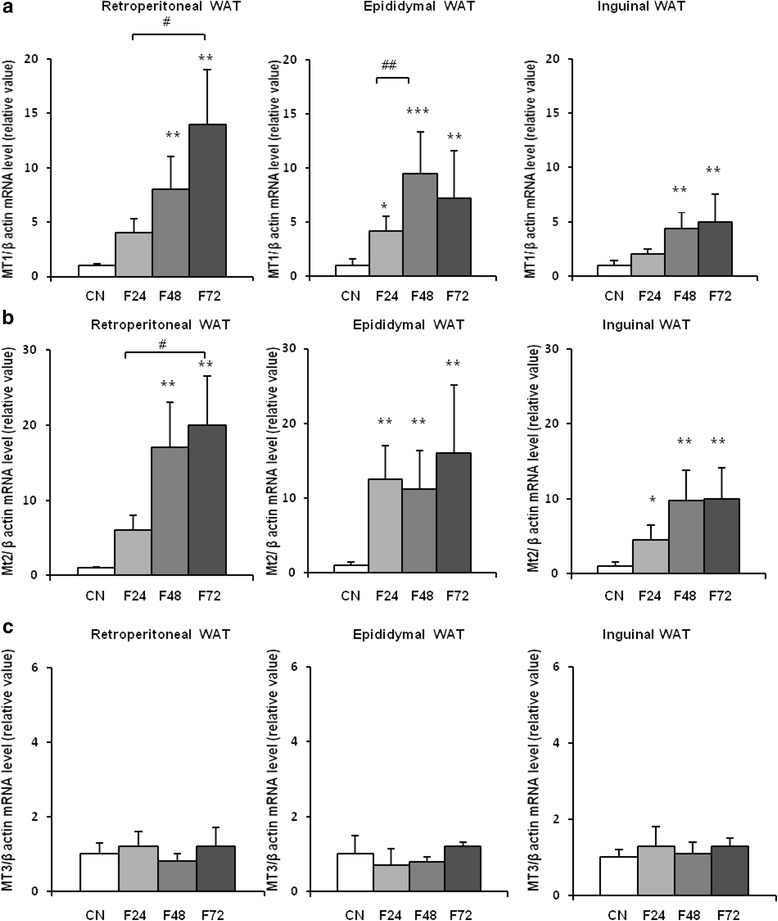
The influence of fasting on MT mRNA level in WAT of rats. MT1 (**a**), MT2 (**b**), and MT3 (**c**) mRNA levels relative to β-actin mRNA levels in retroperitoneal, epididymal, and inguinal WAT of rats fed ad libitum (CN) and fasted rats: 24 h (F24), 48 h (F48), and 72 h (F72) are shown on the graphs (mean ± S.D., *n* = 10) *p < 0.05, **p < 0.01, ***p < 0.001 . Note that MT3 mRNA levels in retroperitoneal, epididymal, and inguinal of control rats were significantly lower than MT1 and MT2

**Fig. 3:**
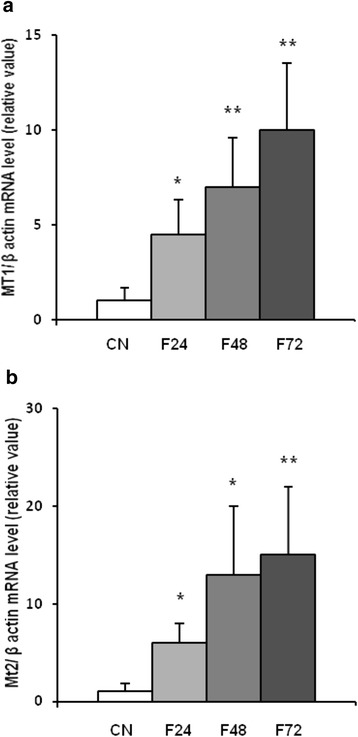
The influence of fasting on MT mRNA level in the liver of rats. MT1 (**a**) and MT2 (**b**) mRNA levels relative to β-actin mRNA levels in the liver of rats fed ad libitum (CN) and fasted rats: 24 h (F24), 48 h (F48), and 72 h (F72) are shown on the graphs (mean ± S.D., *n* = 10) *p < 0.05, **p < 0.01

**Fig. 4:**
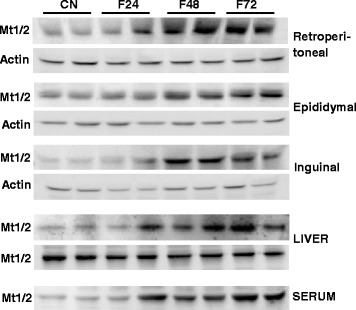
The effect of fasting or fasting/refeeding on MT1 and MT2 (MT1/2) protein level in WAT (retroperitoneal, epididymal, inguinal), liver and serum of rats fed *ad libitum* (CN) and fasted: 24 hours (F24), 48 hours (F48) and 72 hours (F72). Protein levels were assessed by Western blotting (two representative samples from each group are shown). 20 μg of protein per lane was loaded

**Fig. 5:**
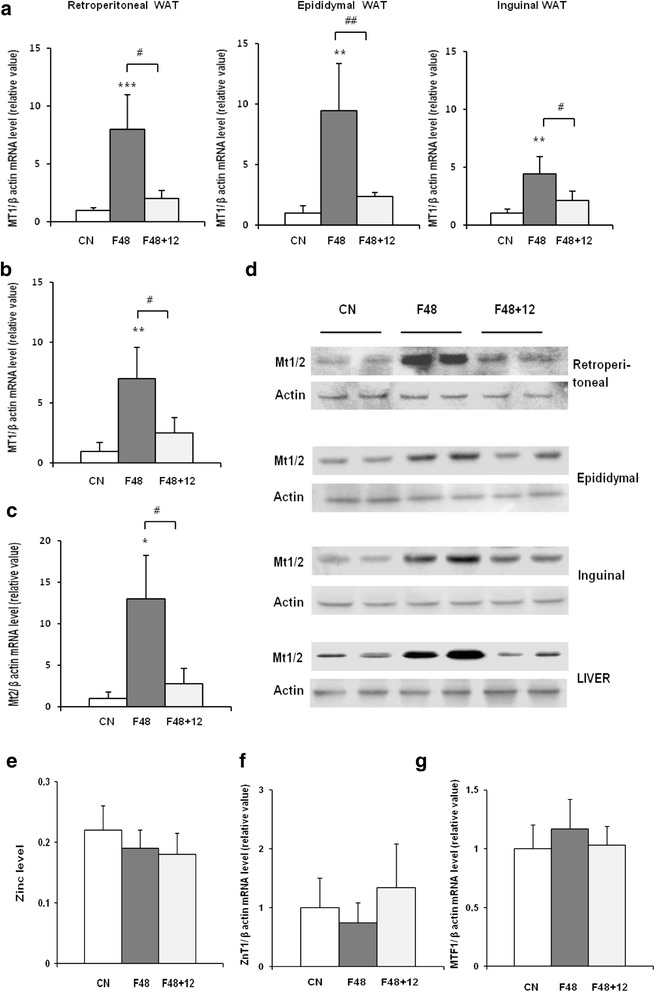
The influence of fasting or fasting/refeeding on MT1 mRNA level in WAT (**a**), liver (**b**, **c**), MT1 and MT2 (MT1/2) protein level in WAT and liver of rats (**d**), zinc level (μg/mg protein) in epididymal WAT (**e**), ZnT1 mRNA level in epididymal WAT (**f**), and MTF1 mRNA level in epididymal WAT(**g**). MT1 mRNA levels relative to β-actin expression in WAT (retroperitoneal, epididymal, and inguinal) and liver of rats fed ad libitum (CN), fasted 48 h (F48), and fasted 48 h and refed 12 h (F48 + 12) are shown on the graphs (mean ± S.D., *n* = 10) *p < 0.05, **p < 0.01, ***p < 0.001; MT1 and MT2 protein level in WAT and liver of rats fed ad libitum (CN), fasted 48 h (F48), and fasted 48 h and refed 12 h (F48 + 12) protein levels were assessed by western blotting (two representative samples from each group are shown, 20 μg of protein per lane was loaded); ZnT1 and MTF1 mRNA levels relative to β-actin mRNA levels in WAT (epididymal) of rats fed ad libitum (CN), fasted 48 h (F48), and fasted 48 h and refed 12 h (F48 + 12) are shown on the graphs (mean ± S.D., *n* = 10)

Using real-time PCR, we detected MT1, MT2, and MT3 mRNA in epididymal, retroperitoneal, and inguinal white adipose tissue (Fig. 1). MT4 mRNA was not found in any of the main fat depots (Fig. 1). The esophagus served as a positive control for MT4 mRNA (Fig. 1). As expected, in the liver, no MT3 mRNA was found (Fig. 1). After a 24-h fast, a significant increase in MT1 (Fig. 2a) and MT2 (Fig. 2b) mRNA levels was observed in epididymal, retroperitoneal, and inguinal WAT. After a 48-h fast, MT1 and MT2 mRNA levels in epididymal and retroperitoneal WAT were several-fold higher than in the controls (Fig. 2a, b). Fasting exerted less prominent effects on MT1 and MT2 mRNA levels in inguinal WAT (Fig. 2a, b), what points to likely differences in fasting response of various WAT depots. The effects of a 72-h fast on MT1 and MT2 mRNA levels in WAT were comparable to those observed after 48 h (Fig. 2a, b). Fasting exerted no significant effects on MT3 mRNA levels in any of the main fat depots (Fig. 2c). An increase in MT1 and MT2 mRNA levels was observed for liver MTs (Fig. 3). The differences in MT1 and MT2 mRNA levels in the WAT and liver of control and fasted rats were reflected by their WAT, liver, and serum MT1 and MT2 protein levels determined by western blot analysis (Fig. 4). When the rats were fed ad libitum after a 48-h fast, their MT1 mRNA levels in retroperitoneal, epididymal, and inguinal WAT were normalized within 12 h (Fig. 5a). Similar effects were also observed for MT2 mRNA levels in retroperitoneal, epididymal, inguinal WAT (Additional file 2: Table S2) and liver MT1 and MT2 (Fig. 5b, c, respectively). Fasting/refeeding exerted no effects on MT3 mRNA levels in any of the main WAT depots (Additional file 2: Table S2). The differences in MT1 and MT2 mRNA levels in the WAT and liver of fasted and refed rats were consistent with the differences in their WAT and liver MT1 and MT2 protein levels determined by western blot analysis (Fig. 5d). Changes in MT1 and MT2 mRNA as well as protein levels apparently were not related to the epididymal WAT total zinc concentrations expressed as μg/mg protein (Fig. 5e) or as μg/g tissue (Additional file 3: Table S3). Moreover, in contrast to MT1 and MT2 mRNA and proteins, ZnT1 mRNA (Fig. 5f) and MTF1 mRNA (Fig. 5g) levels were not affected by fasting or fasting/refeeding. It should be mentioned that others examined Zn transporter mRNAs like ZnT6, ZnT9, ZIP6, ZIP8, and ZIP14 were also not affected by fasting or fasting/refeeding (Additional file 4: Table S4). Thus, the data presented on Fig. 5 suggest that the changes induced by fasting or fasting/refeeding in MT1 and MT2 mRNA levels may be independent of zinc. Body and fat mass (sum of retroperitoneal + epididymal + inguinal WAT) for control (not treated), fasted, and fasted/refed rats are presented in Table 1. Based on data presented in Fig. 2 and Table 1, correlation coefficient between body mass and MT mRNA levels as well between fat mass and MT mRNA levels was calculated. As indicated in Table 2, there was a negative correlation between MT1 mRNA level and body mass or fat mass. Essentially similar correlation for MT2 mRNA was found. As expected, no correlation was revealed in the case of MT3 (Table 2).

**Table 1:** The body and WAT (retroperitoneal, epididymal, inguinal) mass of control, fasted, fasted and refed rats

Group	Body weight (g)	Adipose tissue mass (g)
Control (ad libitum)	261 ± 10	6.4 ± 1.1
Fasted 24 h	227 ± 9***	5.4 ± 1.1*
Fasted 48 h	216 ± 9***	4.9 ± 1.0*
Fasted 72 h	205 ± 8*** ^##^	4.1 ± 0.9* ^#^
Fasted 48 h + ad libitum	242 ± 9* ^##^	4.7 ± 1.4**

The body mass and adipose tissue of rats maintained on fasting and fasting and refeeding (mean ± S.D., *n* = 10). *p < 0.05, **p < 0.01, ***p < 0.001 compared to the control group, ^#^ *p* < 0.05, ^##^ *p* < 0.01 compared to 24-h fasted group

**Table 2:** Correlation coefficients between body weight and MT mRNA level in retroperitoneal, epididymal, or inguinal WAT and the sum of adipose tissue mass and MT mRNA relative level in retroperitoneal, epididymal, or inguinal WAT

WAT	mRNA	Body weight		Adipose tissue mass	
		*R*	*p*	*R*	*p*
Retroperitoneal	MT1	−0.60	<0.001	−0.63	<0.001
	MT2	−0.54	<0.01	−0.52	<0.01
	MT3	0.12	ns	0.02	ns
Epididymal	MT1	−0.66	<0.001	−0.48	<0.01
	MT2	−0.52	<0.01	−0.43	<0.02
	MT3	−0.12	ns	−0.05	ns
Inguinal	MT1	−0.82	<0.001	−0.66	<0.001
	MT2	−0.83	<0.001	−0.54	<0.01
	MT3	0.18	ns	0.12	ns

*R* correlation coefficients; *ns* non-significant

**Fig. 6:**
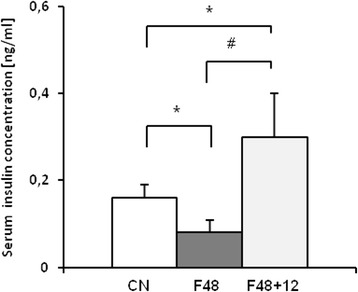
The influence of fasting and fasting/refeeding on insulin concentration in rat serum. Insulin concentration in serum of rats fed ad libitum (CN), fasted 48 h (F48), and fasted 48 h and refed 12 h (F48 + 12) are shown on the graph (mean ± S.D., *n* = 10) *p < 0.05

**Fig. 7:**
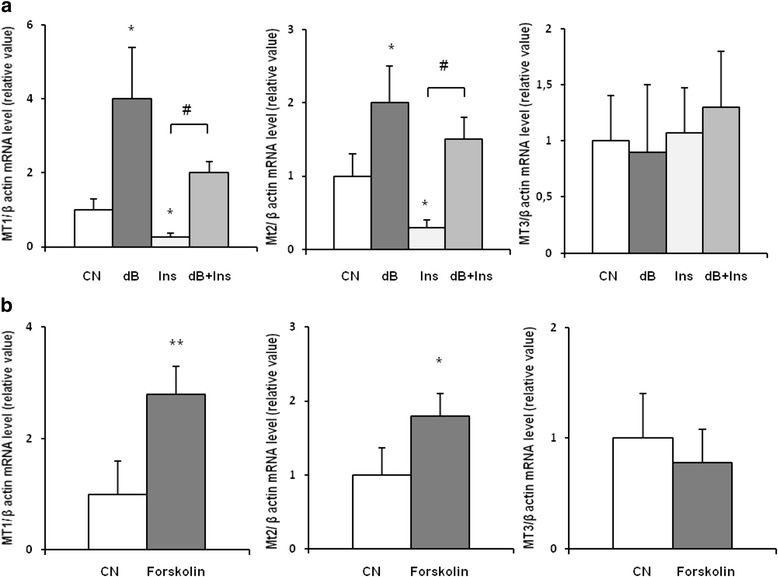
The effect of insulin (Ins) and dB-cAMP (dB) (**a**) and forskolin (**b**) in primary rat adipocytes on MTs mRNA expression. MT1, MT2, and MT3 mRNA level relative to β-actin mRNA levels in primary rat adipocytes: control (CN), dB-cAMP, insulin, dB-cAMP plus insulin, and forskolin-treated adipocytes are shown on the graph (mean ± S.D., *n* = 5) *p < 0.05,**p < 0.01

To obtain more information about potential factor(s) that may contribute to food intake-related changes in MT1 and MT2 gene expressions, we determined serum concentrations of insulin in control, fasted, and refed rats. As shown in Fig. 6, fasting was associated with a significant decrease in serum insulin, whereas refeeding resulted in an increase of this parameter. An inverse association between serum insulin and MT1 and MT2 mRNA levels in WAT suggests that insulin may influence, at least partially the expressions of MT1 and MT2 encoding genes. To verify this hypothesis, we studied the effects of insulin on MT1 and MT2 levels in isolated adipocytes from epididymal WAT. As shown in Fig. 7, exposure of adipocytes to insulin (which theoretically should mimic a fed state) was reflected by a significant decrease in MT1 mRNA level. When dB-cAMP was added to the primary culture (which theoretically should mimic a fasted state), a significant increase in MT1 mRNA level was observed (Fig. 7a). Further, addition of insulin to adipocytes incubated in the presence of dB-cAMP was reflected by a significant decrease in MT1 mRNA levels. Essentially, similar results were obtained for MT2 mRNA level (Fig. 7a). Incubation with insulin or/and dB-cAMP exerted no effects on MT3 mRNA level in the primary culture of isolated adipocytes (Fig. 7a). Moreover, MT1 and MT2 mRNA in isolated rat adipocytes was also upregulated by forskolin (Fig. 7b), a compound increasing the intracellular cAMP. No significant effect of forskolin on MT3 mRNA level was found (Fig. 7b).

## Discussion

Our present study showed that (a) MT1, MT2, and MT3 isoforms, but not MT4, are expressed in all main fat depots (retroperitoneal, epididymal, and inguinal) of rats, (b) MT1 and MT2 encoding genes are overexpressed in all main fat depots of fasted rats, (c) refeeding of fasted rats leads to a decrease in the expressions of MT1 and MT2 encoding genes in WAT, and (d) expression of MT3 encoding gene in retroperitoneal, epididymal, and inguinal WAT is not affected by fasting/refeeding. The magnitude of induction of MT1 and MT2 mRNA (and protein) levels is dependent on adipose tissue localization, since fasting exerted less prominent effects on MT1 and MT2 mRNA levels in inguinal (subcutaneous) than in epididymal and retroperitoneal WAT (Fig. 2a, b). The differences between MT levels in omental and subcutaneous human adipose tissue were also reported by Kim et al. [23]. Furthermore, our data imply that the effects of fasting on MT1 and MT2 mRNA levels in WAT are generally similar to those observed in the liver (Figs. 2 and 3). Thus, the effect of fasting on the liver content of MTs presented here confirms the results reported previously by Higashimoto et al. [17] and Kondoh et al. [25] in mice.

Our findings differ considerably from those reported previously by Trayhurn et al. [44] according to whom MT1 mRNA levels in epididymal WAT of fasted and fasted/refed mice were similar to the controls. In contrast, our fasted rats presented significantly higher MT1 and MT2 mRNA levels than the control animals. Moreover, we showed that the effects of fasting on MT mRNA levels are time-dependent, reaching its peak after a 48-h fast. Thus, one possible explanation for the discrepancy between the data published by Trayhurn et al. [43] and the results of our study (beside different animal model used) may be a duration of fasting. In Trayhurn et al.’s [43] experiment, mice were fasted for up to 16 h. As shown in Fig. 2, the level of MT1 mRNA (as well as MT2 RNA) in epididymal WAT increased approximately twofold after 24-h fast and several fold after 48 and 72 h fast compare to the control.

The effects of fasting on the expressions of MT1 and MT2 encoding genes in the liver and WAT (as well as on the serum concentrations of MT1 and MT2), measured at both mRNA and protein level, were completely reversed after a 12-h refeeding ad libitum. This implies that the expressions of MT1 and MT2 encoding genes in all main fat depots of rats are highly sensitive to nutritional status. Consequently, the levels of MT1 and MT2 in WAT and blood slightly fluctuate throughout the day in response to food intake.

Interestingly, the results presented here indicate that the expression of genes encoding MT1 and MT2 regulated by fasting and fasting/refeeding could be independent of total zinc content in WAT. Induction of gene encoding MT1 expression in rat brown adipose tissue [32] and mouse liver [13] independent of zinc has been also reported previously. However, regarding the fact that intracellular zinc homeostasis is regulated by MTs [8, 26, 30], overexpression of genes coding MTs in fasted rats and subsequently the increase in MTs levels could decrease free zinc ion (but not total zinc levels) concentration in adipocytes necessary for zinc finger protein biosynthesis. This may affect zinc finger protein biosynthesis and subsequently, adipose tissue function [48]. It is well documented that the zinc finger proteins regulate adipogenesis and lipid metabolism in adipose tissue [48]. Thus, one may suppose that increased MT1 and MT2 levels may lead to decrease in intracellular zinc finger protein levels and subsequently to inhibition of adipogenesis and lipid accumulation in adipose tissue. Diminished levels of intracellular MTs may lead to the increase of zinc finger protein levels and subsequently to the activation of adipogenesis and increase in lipid accumulation in adipose tissue, which in turn may lead to obesity. Accordingly, the data reported previously indicate that MTs (1 and 2) null mice are obese [2, 37].

It should be noted that besides MTs, intracellular zinc homeostasis may be regulated by zinc transporters, SLC30A (also named ZnT) and SLC39A (also named ZIP) [42]. However, the results presented in this paper indicate no association between MTs and Zn transporter mRNA levels.

Moreover, we showed that a 24-h incubation with dB-cAMP (or forskolin) results in increase, whereas a 24-h exposure to insulin leads to decrease in MT1 and MT2 mRNA levels in isolated adipocytes (Fig. 7). Thus, the effect of cAMP on MT gene expression in isolated rat adipocytes is similar to that reported by Trayhurn [44]. However, in contrast to our results, Trayhurn et al. [44] found increase in MT gene expressions in rat adipocytes incubated with insulin. Based on the data indicating that insulin leads to decrease of cAMP concentrations in adipocytes [12], the data reported by Trayhurn et al. [44] are difficult to explain. Moreover, our results are in line with data reported by Costarelli et al. [9], who found inverse relationship between human plasma insulin and MT concentrations.

Taken together, the results of our study imply that nutritional status plays a key role in the regulation of MT1 and MT2 encoding genes in WAT. Inverse relationship between serum insulin concentration and MTs (1 and 2) mRNA levels in WAT (Figs. 5a and 6) as well as the decrease in MT1 and MT2 mRNA levels by insulin in isolated adipocytes (Fig. 7) suggests that the effect of nutritional status on MT1 and MT2 is mediated by insulin. Previous studies showed that MT promoter has the sequence for AP-2 (activating protein-2), a transcription factor via which cAMP upregulates mRNA level in the liver [10]. Insulin is likely to suppress MT1 and MT2 encoding genes upon binding to adipocyte insulin receptors and their activation; this results in the phosphorylation of insulin receptor substrates, activation of the phosphatidylinositol kinase 3 (PI3K)-kinase B/Akt (PKB/Akt)-phosphodiesterase 3B pathway, and degradation of cAMP in adipocytes [12]. These events lead to a decrease in the intracellular concentrations of cAMP and resultant decrease in MT1 and MT2 mRNA levels. Consequently, both lack of insulin and insulin insensitivity may upregulate MT1 and MT2 encoding genes in WAT of rats. However, it is not excluded that insulin stimulated glucose influx into adipocytes could be responsible for the inhibition of gene expression encoding MT1 and MT2 in WAT. Although our experimental data cannot be directly extrapolated to humans, the previously reported increase in MT2a gene expression in the adipose tissue from type 2 diabetic patients [16] and obese subject [7, 11] might be at least in part associated with insulin insensitivity.

Considering that glucocorticosteroids stimulate the expression of gene encoding MT1 and MT2 in the liver [24], one can suppose that fasting-induced increase in these steroid levels [49] may also at least in part contribute to the observed upregulation of MTs (1 and 2) in WAT and liver in vivo.

According to Trayhurn et al. [43], MT is a secretory protein synthesized in WAT (though the primary structure of MT is inconsistent with that of the classic secretory protein), and as such, may exert systemic biological effects. Our presented findings imply that enhanced expressions of MT1 and MT2 genes in WAT and liver (observed at both mRNA and protein levels) correlate strongly with an increase in the serum concentrations of MT1 and MT2 (Fig. 4). Therefore, liver and WAT might contribute to elevated serum MT levels. Consequently, the results of our study seem to support the hypothesis that MTs are secretory products of WAT, as proposed by Trayhurn et al. [43]. However, it is not excluded that some amount of MTs are released from blood cells during the preparation of serum.

## Conclusions

In conclusion, our findings imply that the genes encoding MT1 and MT2, but not MT3, in retroperitoneal, epididymal, and inguinal WAT of rats are regulated by nutritional status and that insulin plays an important role in this process. Changes in MT1 and MT2 mRNA levels were not related to (a) total zinc concentrations, (b) MTF1 mRNA level, and (c) Zn transporters mRNA levels in WAT. This suggests that the regulation of MT1 and MT2 gene expression by fasting and fasting/refeeding may be independent of total zinc concentrations. It is likely that changes in WAT MT1 and MT2 levels induced by nutritional status could be an important system to regulate intracellular distribution, but not total concentrations of zinc.

## Abbreviations

cAMP, cyclic adenosine monophosphate; Cu, copper; dB-cAMP, dibutyryl cyclic adenosine monophosphate; DMEM, Dulbecco’s Modified Eagle’s Medium; HEPES, *N*-(2-Hydroxyethyl)piperazine-*N*′-(2-ethanesulfonic acid); MT, metallothionein; MTF1, metal-responsive transcription factor; PCR, polymerase chain reaction; RNA, ribonucleic acid; SD, standard deviations; TBP, TATA-box binding protein; WAT, white adipose tissue; Zn, zinc

## Additional files

Additional file 1: Table S1. Primer sequences for real time PCR. (DOCX 14 kb) Additional file 2: Table S2. The influence of fasting or fasting/refeeding on: MT2 and MT3 mRNA level relative to β-actin expression in WAT of rats. (DOCX 13 kb) Additional file 3: Table S3. Zinc concentration in epididymal WAT. (DOCX 11 kb) Additional file 4: Table S4. The influence of fasting or fasting/refeeding on: ZnT6, ZnT9, ZIP6, ZIP9 and ZIP14 mRNA level relative to β-actin expression in epididymal WAT of rats. (DOCX 13 kb)
