# Supplementary material for: Metallothioneins 1 and 2, but not 3, are regulated by nutritional status in rat white adipose tissue
Source: Genes Nutr. 2016 Jun 23;11:18. doi: 10.1186/s12263-016-0533-3 (PMC4968437; doi:10.1186/s12263-016-0533-3)
Supplement: Additional file 1: Table S1. — Primer sequences for real time PCR. (DOCX 14 kb) [file 12263_2016_533_MOESM1_ESM.docx]

| **Gene** | **Forward (F) and reverse (R) primer sequence** |
| --- | --- |
| β-actin | F: 5′ - TGTCACCAACTGACGATA - 3′. R: 5 ′- GGGGTGTTGAAGGTCTCAAA - 3′ |
| TBP | F: 5’ - CACCGTGAATCTTGGCTGTAAAC - 3’ R: 5’ - ATGATGACTGCAGCAAACCG - 3’ |
| cyclophilin A | F: 5’ – CTGAGCACTGGGGAGAAAGGA – 3’ R: 5’ – GAAGTCACCACCCTGGCACA – 3’ |
| Mt1 | F: 5′ - CCCGTGGGCTGCTCCAAATGT - 3′ R: 5′ - ACTGGGTGGAGGTGTACGGCA - 3′ |
| Mt2 | F: 5′ - GCGATCTCTCGTTGATCTCC - 3′ R: 5′ - CAGGAGCAGGATCCATCTGT - 3′ |
| Mt3 | F: 5’ - TGGTTCCTGCACCTGCTCGG - 3′ R: 5′ - TGGGAGTCCTCACTGGCAGCA - 3′ |
| Mt4 | F: 5′ - ACACACCTGGACCATGGACCCT - 3′ R: 5′ - AGCAGGGGCAGCAGCTTTTACG - 3′ |
| MTF1 | F: 5’ - ACCAAGAACAAATTCAGCAAGCA - 3’ R: 5’ - CACTGACAGGCCTCCTCTTG - 3’ |
| ZnT1 | F: 5′ - CACCGTGAATCTTGGCTGTAAAC - 3′ R: 5′ - ATGATGACTGCAGCAAACCG - 3′ |
| ZnT6 | F: 5’- GCCTGAGATACACACGGGAA - 3’ R: 5’ - GCGACTAAGGTCTGCCACAT - 3’ |
| ZnT9 | F: 5’ - GCAGCCAGGGATGGAAGAAT - 3’ R: 5’ - TATTTGCGGGTGAATGCTGG - 3’ |
| ZIP6 | F: 5’ - CGGGAGACGCACCAAAGATA - 3’ R: 5’ - TGTTGCATTGAAGCACTCCTG - 3’ |
| ZIP8 | F: 5’ - TCCTTTTATCTCAGGCTCCGC - 3’ R: 5’ - CTGCCCCAGTTAGCGAGAAG - 3’ |
| ZIP14 | F: 5’ - CATTGAAGTGTGGGGCTTTGG-3’ R: 5’ - CCAATGGACAAGGCGATGAAG - 3’ |

**Additional file 1: Table S1** Primer sequences for real time PCR
