# Supplementary material for: Metallothioneins 1 and 2, but not 3, are regulated by nutritional status in rat white adipose tissue
Source: Genes Nutr. 2016 Jun 23;11:18. doi: 10.1186/s12263-016-0533-3 (PMC4968437; doi:10.1186/s12263-016-0533-3)
Supplement: Additional file 2: Table S2. — The influence of fasting or fasting/refeeding on: MT2 and MT3 mRNA level relative to β-actin expression in WAT of rats. (DOCX 13 kb) [file 12263_2016_533_MOESM2_ESM.docx]

**Additional file 2: Table S2** The influence of fasting or fasting/refeeding on: MT2 and MT3 mRNA level relative to β-actin expression in WAT of rats

|  | | **WAT** | | | | | | | | |
| --- | --- | --- | --- | --- | --- | --- | --- | --- | --- | --- |
|  | | **epididymal** | | | **retroperitoneal** | | | **inguinal** | | |
|  |  | **CN** | **F48** | **F48+12** | **CN** | **F48** | **F48+12** | **CN** | **F48** | **F48+12** |
| **MT2** | **mean** | 1 | 11.5 ^**^ | 1.17 ^#^ | 1 | 17 ^**^ | 1.27 ^#^ | 1 | 9.7 ^**^ | 1.32 ^#^ |
|  | **S.D.** | 0.11 | 5,2 | 0,29 | 0.08 | 7.2 | 0.24 | 0.13 | 3.31 | 0.41 |
| **MT3** | **mean** | 1 | 0.9 ^ns^ | 1.15 ^ns^ | 1 | 0.83 ^ns^ | 0.95 ^ns^ | 1 | 1.03 ^ns^ | 1.15 ^ns^ |
|  | **S.D.** | 0.38 | 0.12 | 0.26 | 0.23 | 0.31 | 0.18 | 0.17 | 0.19 | 0.21 |

(CN) fed *ad libitum*, (F48) fasted 48 hours, (F48+12) fasted 48 hours and refed 12 hours; n = 10; ** p < 0.01 compared to the control group,
 # p < 0.05 compared to 48 hours fasted group; ns - not significant
