# Supplementary material for: Metallothioneins 1 and 2, but not 3, are regulated by nutritional status in rat white adipose tissue
Source: Genes Nutr. 2016 Jun 23;11:18. doi: 10.1186/s12263-016-0533-3 (PMC4968437; doi:10.1186/s12263-016-0533-3)
Supplement: Additional file 3: Table S3. — Zinc concentration in epididymal WAT. (DOCX 11 kb) [file 12263_2016_533_MOESM3_ESM.docx]

**Additional file 3: Table S3** Zinc concentration in epididymal WAT

|  | **Zn [µg/g tissue]** | | |
| --- | --- | --- | --- |
|  | **CN** | **F48** | **F48+12** |
| mean | 2.34 | 2.44 ^ns^ | 2.52 ^ns^ |
| S.D. | 0.42 | 0.39 | 0.29 |

ns - non-significant
