# Supplementary material for: Metallothioneins 1 and 2, but not 3, are regulated by nutritional status in rat white adipose tissue
Source: Genes Nutr. 2016 Jun 23;11:18. doi: 10.1186/s12263-016-0533-3 (PMC4968437; doi:10.1186/s12263-016-0533-3)
Supplement: Additional file 4: Table S4. — The influence of fasting or fasting/refeeding on: ZnT6, ZnT9, ZIP6, ZIP9 and ZIP14 mRNA level relative to β-actin expression in epididymal WAT of rats. (DOCX 13 kb) [file 12263_2016_533_MOESM4_ESM.docx]

**Additional file 4: Table S4** The influence of fasting or fasting/refeeding on: ZnT6,
ZnT9, ZIP6, ZIP9 and ZIP14 mRNA level relative to β-actin
expression in epididymal WAT of rats

|  |  | **CN** | **F48** | **F48+12** |
| --- | --- | --- | --- | --- |
| **ZnT6** | mean | 1 | 1.04 ^ns^ | 1.03 ^ns^ |
|  | S.D. | 0.42 | 0.31 | 0.21 |
| **ZnT9** | mean | 1 | 1.21 ^ns^ | 1.17 ^ns^ |
|  | S.D. | 0.35 | 0.34 | 0.11 |
| **ZIP6** | mean | 1 | 1.24 ^ns^ | 0.98 ^ns^ |
|  | S.D. | 0.17 | 0.45 | 0.26 |
| **ZIP9** | mean | 1 | 0.87 ^ns^ | 0.75 ^ns^ |
|  | S.D. | 0.49 | 0.39 | 0.28 |
| **ZIP14** | mean | 1 | 0.89 ^ns^ | 1.03 ^ns^ |
|  | S.D. | 0.41 | 0.45 ^ns^ | 0.23 ^ns^ |

(CN) fed *ad libitum*; (F48) fasted 48 hours; (F48+12) fasted 48 hours and refed 12 hours; n = 10; ns - non-significant
